# Prenatal maternal infections and early childhood development outcomes: An analysis of Scottish linked administrative health data

**DOI:** 10.23889/ijpds.v8i2.2222

**Published:** 2023-09-14

**Authors:** Iain Hardie, Aja Murray, Josiah King, Michael Lombardo, Philip Wilson, Louise Marryat, Lucy Thompson, Helen Minnis, Bonnie Auyeung

**Affiliations:** 1 Department of Psychology, School of Philosophy, Psychology and Language Sciences, The University of Edinburgh, Edinburgh, United Kingdom; 2 Laboratory for Autism and Neurodevelopmental Disorders, Center for Neuroscience and Cognitive Systems, Istituto Italiano di Tecnologia, Rovereto, Italy; 3 Centre for Rural Health, Institute of Applied Health Sciences, University of Aberdeen, Aberdeen, United Kingdom; 4 School of Health Sciences, University of Dundee, Dundee, United Kingdom; 5 School of Health and Wellbeing, University of Glasgow, Glasgow, United Kingdom

## Objectives

Previous research suggests that prenatal maternal infections may be linked to later childhood neurodevelopmental outcomes and socioemotional difficulties. We exploited a large linked administrative health dataset to examine relationships between prenatal infections and early childhood development outcomes in Greater Glasgow & Clyde (GGC), Scotland.

## Method

We used population data from birth records, hospital records, prescriptions and routine child health reviews for 55,856 children born in GGC, 2011-2015, and their mothers. Logistic regression models were used to examine the relationship between prenatal infections, measured as both hospital-diagnosed prenatal infections and receipt of infection-related prescription(s) during pregnancy, and having adverse childhood development outcomes identified by health visitors during 6-8 weeks/27-30 months routine child health reviews. Secondary analysis examined whether results varied by (a) specific development outcome types (i.e. gross-motor-skills, hearing-communication, vision-social-awareness, personal-social, emotional-behavioural-attention, and speech-language-communication development), and (b) the trimester(s) in which infections occurred.

## Results

After adjusting for confounders/covariates, hospital-diagnosed infections were associated with increased odds of having at least one adverse development outcome identified during child health reviews (OR: 1.30; 95% CI: 1.19-1.42). This relationship was consistent across almost all development outcome types, and appeared to be specifically linked to infections occurring in trimesters 2 (OR: 1.34; 95% CI: 1.07-1.67) and 3 (OR: 1.33; 95% CI: 1.21-1.47), i.e. the trimesters in which fetal brain myelination occurs. Infection-related prescriptions were not associated with a significant increase in odds of having at least one adverse development outcome after adjusting for confounders/covariates (OR: 1.03; 95% CI: 0.98-1.08), but were associated with slightly increased odds of adverse outcomes specifically related to personal-social (OR: 1.12; 95% CI: 1.03-1.22) and emotional-behavioural-attention (OR: 1.15; 95% CI: 1.08-1.22) development.

## Conclusion

Prenatal infections, particularly those which are hospital-diagnosed and therefore likely to be more severe, are associated with early childhood development outcomes. Our study highlights the usefulness of Scotland’s administrative health data in measuring childhood development. Future research will examine the impact of COVID-19 prenatal infections and lockdown measures.

